# Crystal structure of tetra­methyl­ammonium 1,1,7,7-tetra­cyano­hepta-2,4,6-trienide

**DOI:** 10.1107/S2056989019011411

**Published:** 2019-08-23

**Authors:** Georgii Bogdanov, John P Tillotson, Jenna Bustos, Marina Fonari, Tatiana V. Timofeeva

**Affiliations:** a Department of Chemistry, New Mexico Highlands University, Las Vegas, New Mexico, 87701, USA; b School of Chemistry and Biochemistry, Georgia Institute of Technology, Atlanta, Georgia, 30332, USA

**Keywords:** crystal structure, polymethines, carbanion, hepta-2,4,6-triene-1,1,7,7-tetra­carbo­nitrile, stacking, weak inter­actions

## Abstract

The title compound, C_4_H_12_N^+^·C_11_H_5_N_4_^−^, contains one tetra­methyl­ammonium cation and one 1,1,7,7-tetra­cyano­hepta-2,4,6-trienide anion in the asymmetric unit. The anion is in an all-*trans* conjugated C=C bonds conformation. Two terminal C(CN)_2_ di­nitrile moieties are slightly twisted from the polymethine main chain to which they are attached [C(CN)_2_/C_5_ dihedral angles = 6.1 (2) and 7.1 (1)°]. The C—C bond distances along the hepta­dienyl chain vary in the narrow range 1.382 (2)–1.394 (2) Å, thus indicating the significant degree of conjugation. In the crystal, the anions are linked into zigzag chains along the [10] direction by C—H⋯N(nitrile) short contacts. The anti­parallel chains stack along the [110] direction with alternating separations between the neighboring anions in stacks of 3.291 and 3.504 Å. The C—H⋯N short contacts and stacking inter­actions combine to link the anions into layers parallel to the (01) plane and separated by columns of tetra­methyl­ammonium cations.

## Abstract 2

In the crystal, C—H⋯N(nitrile) short contacts and stacking inter­actions combine to link the anions into layers parallel to the (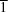
01) plane and separated by columns of tetra­methyl­ammonium cations.

## Chemical context   

Polymethines, being fully conjugated hydro­carbons, represent the simplest ‘mol­ecular wires’ with potential uses in organic electronic applications thanks to their easily tuned band gaps, and their wide range of absorption covering the visible spectrum (Etemad & Heeger, 1982 ▸; Meisner *et al.*, 2012 ▸; Jayamurugan *et al.*, 2014 ▸). Crystallographic data for polymethines are rather scare because of their instability and low solubility (Chetkina & Bel’skii, 2002 ▸; Meisner *et al.*, 2012 ▸; Tsuji & Hoffmann, 2016 ▸). A successful strategy to increase the chemical stability with respect to oxidative decomposition has been reported (Meisner *et al.*, 2012 ▸) that includes the decoration of polyenes with cyano groups and which resulted in the synthesis of a library of odd-numbered members from three to thirteen linear conjugated olefins and the determination of their crystal structures.
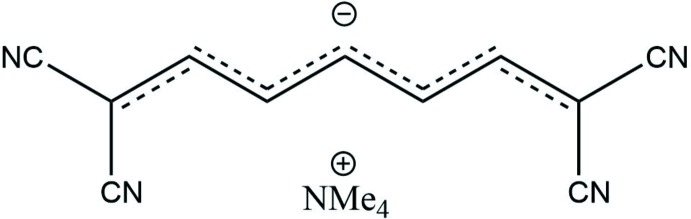


## Structural commentary   

**Figure 1:**
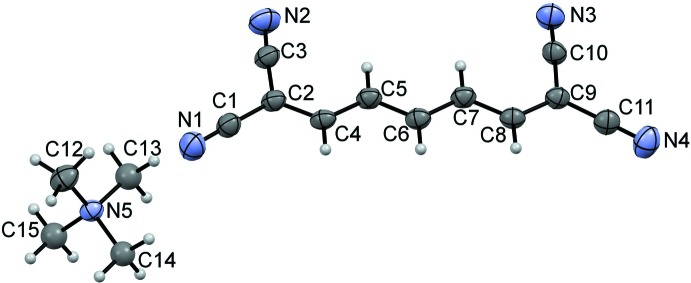
The formula unit of the title compound with the atom labelling. Displacement ellipsoids are drawn at the 50% probability level. Only the major components of the disordered methyl groups in the cation are shown.

The title compound (Fig. 1 ▸) crystallizes with one cation and one anion per asymmetric unit, both entities residing in general positions. The trimethyl­ammonium cation has a common tetra­hedral geometry (2460 hits for this cation in CSD version 5.40, last update November 2018; Groom *et al.*, 2016 ▸), with three of the four methyl groups being disordered (see *Refinement*). In the linear anion, the bond lengths vary in the narrow range 1.382 (2)–1.394 (2) Å, thus indicating a significant degree of conjugation along the hydro­carbon chain. Such a structural and electronic configuration in which the difference in bond lengths along the conjugated backbone approaches zero is known as a cyanine-like structure (Marder *et al.*, 1994 ▸). The anion is slightly distorted from a planar arrangement as shown by the r.m.s. deviation of 0.098 Å for non-hydrogen atoms from the least-square plane calculated through the entire carbanion. The dihedral angles between the perfectly planar terminal di­cyano-groups, C(CN)_2_ and the linear C4–C8 central fragment in the anion are 6.1 (2) and 7.1 (1)°. The bond lengths and angles and the overall conformation of the anion are close to those reported for the same anion in *N*-(7-(di­methyl­amino)­hepta-2,4,6-trienyl­idene)-*N*,*N*-di­methyl­ammonium 1,1,7,7-tetra­cyano­hepta-2,4,6-trienide (NEQHOH; Reck & Dahne, 2006 ▸), and for its di­cyano derivative, 1,1,2,6,7,7-hexa­cyano­hepta­trienide in the ammonium salt (Edmonds *et al.*, 1970 ▸).

## Supra­molecular features   

**Figure 2:**
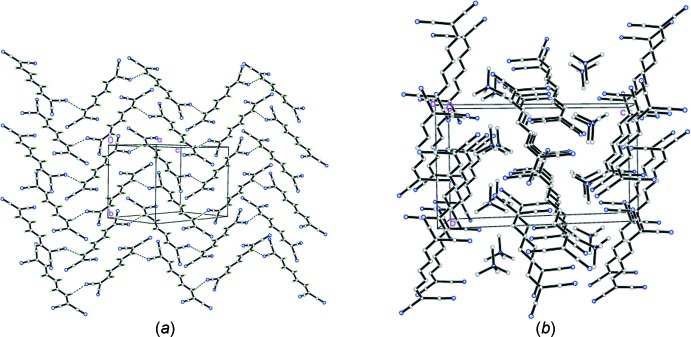
The crystal packing in the title compound showing (*a*) supra­molecular anionic chains with C—H⋯N inter­actions packed in a layer parallel to the (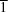
01) plane and (*b*) the packing. The minor disorder component is omitted for clarity.

In the crystal, anions related by the twofold screw axis are linked by C4—H4⋯N3^i^ short contacts (Table 1 ▸), forming zigzag chains along the [10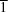] direction in which adjacent mol­ecules have a nearly orthogonal arrangement, as indicated by the dihedral angle between their skeletons of 87.62°. The anti­parallel chains stack along the [110] direction with alternating separations between neighboring anions in the stacks of 3.291 and 3.504 Å. The C—H⋯N short contacts (Table 1 ▸) and stacking inter­actions of 3.291 and 3.504 Å combine to form layers of anions parallel to the (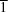
01) plane and separated by columns of tetra­methyl­ammonium cations (Fig. 2 ▸). A similar arrangement with separation of the anionic and cationic regions was noted in the crystal structure of tetra­methyl­ammonium 1,1,2,4,5,5-hexa­cyano­penta­dienide (HXCPEN; Sass & Nichols, 1974 ▸).

## Database survey   

**Figure 3:**
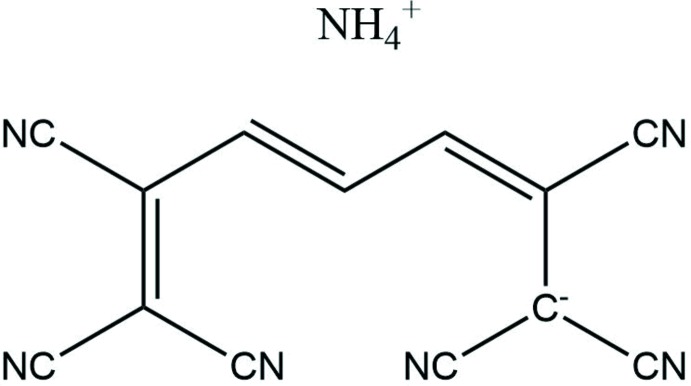
Chemical structure of AHCNPI.

**Figure 4:**
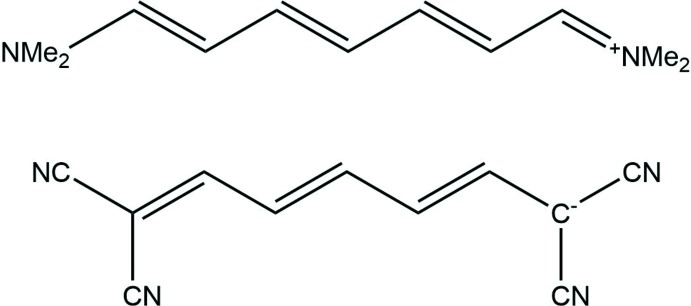
Chemical structure of NEQHOH.

The Cambridge Structural Database (CSD version 5.40, last update November 18, Groom *et al.*, 2016 ▸) provides very scare solid-state structural information on linear oligoenes, a search for linear tetra­cyano­hepta­trienide analogues of the title compound yielding only two structures: ammonium 1,1,2,6,7,7-hexa­cyano­hepta­trienide (AHCNPI; Edmonds *et al.*, 1970 ▸) and *N*-(7-(di­methyl­amino)­hepta-2,4,6-trienyl­idene)-*N*,*N*-di­methyl­ammonium 1,1,7,7-tetra­cyano­hepta-2,4,6-trienide (NEQHOH; Reck & Dahne, 2006 ▸). The reported room-temperature data revealed the similar values for the bond lengths along the hepta­trienide backbone, which are in the range 1.378–1.390 Å in AHCNPI (Fig. 3 ▸) and 1.368 (5)–1.389 (5) Å in NEQHOH (Fig. 4 ▸). For the two nearest homologues of the title compound with six and eight carbon atoms in the main chains, seven hits (DBPHCN and PHXTCN; Noerenberg *et al.*, 1977 ▸; QAGXUU, QAGYAB, QAGYEF, QAGYIJ and QAGYOP; Jayamurugan *et al.*, 2014 ▸) and one (QAGXOO, Jayamurugan *et al.*, 2014 ▸) hit were found in the CSD, all of which represent individual mol­ecules decorated by either phenyl or nitrile substituents.

## Synthesis and crystallization   

**Figure 5:**
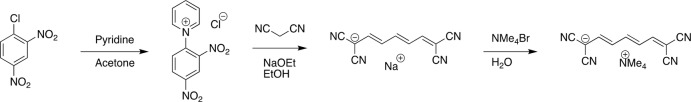
Synthesis of the title compound.

The synthesis is shown in Fig. 5 ▸.

***N*** **-(2,4-di­nitro­phen­yl)pyridinium chloride.** Pyridine (4.00 mL, 49.4 mmol) was added to a solution of 2,4-di­nitro­chloro­benzene (10.00 g, 49.37 mmol) in dry acetone (4 mL). The resulting mixture was brought to reflux for 1 h before being cooled to room temperature. The crude product was collected by filtration and recrystallized from ethanol to give *N*-(2,4-di­nitro­phen­yl)pyridinium chloride (11.26 g, 91%), m.p. 462–463 K. ^1^H NMR (500 MHz, CDCl_3_) δ 9.38 (*d*, *J* = 5.3 Hz, 2H), 9.12 (*d*, *J* = 2.3 Hz, 1H), 8.99 (*dd*, *J* = 8.2, 2.3 Hz, 1H), 8.95 (*t*, *J* = 8.0 Hz, 1H), 8.44 (*m*, 3H).

**Tetra­methyl­ammonium (2** ***E*** **,4** ***E*** **)-1,1,7,7-tetra­cyano­hepta-2,4,6-trien-1-ide.** Malono­nitrile (0.42 g, 6.3 mmol) was added to a refluxing solution of fresh sodium ethoxide, prepared by adding sodium metal (0.20 g, 8.7 mmol) to absolute ethanol (5 mL). To this solution, was added a solution of *N*-(2,4-di­nitro­phen­yl)pyridinium chloride (0.71 g, 2.5 mmol) in ethanol (2 mL), and the reaction mixture was stirred at reflux for 1 h before being cooled to room temperature and stirred for a further hour. A solution of tetra­methyl­ammonium bromide (0.39 g, 2.5 mmol) in water (10 mL) was added to the reaction. After about an hour, the deep-red mixture was extracted with di­chloro­methane (3 × 30 mL), dried over magnesium sulfate, and the solvent was removed *in vacuo*. The deep-violet residue was purified by column chromatography (silica gel, 10% acetone in CHCl2). ^1^H NMR (500 MHz, CDCl_3_) δ 7.06 (*d*, *J* = 12.9 Hz, 2H), 6.95 (*t*, *J* = 12.9 Hz, 1H), 6.06 (*t*, *J* = 12.8 Hz, 2H), 3.68 (*s*, 12H).

**Crystallization**. Crystals of the title compound were grown over a period of 2–4 weeks by the vapour-diffusion method using di­chloro­methane as the solvent and hexane as the non-solvent.

## Refinement   

Crystal data, data collection and structure refinement details are summarized in Table 2 ▸. C-bound H atoms were fixed geometrically (C—H = 0.95–0.98 Å) and refined using a riding model, with *U*_iso_(H) set to 1.2*U*_eq_(C) for aromatic and 1.5*U*_eq_(C-meth­yl). To obtain an idealized geometry of the cation, 1,2 and 1,3 restraints for C—N mean bond distances and C—N—C bond angles were used. In the tetra­methyl ammonium cation, three methyl groups are each disordered over two positions about the N5—C12 axis and were refined with partial occupancies of 0.66 (1) and 0.34 (1). The positions of all disordered atoms were refined in an isotropic approximation.

## Supplementary Material

Crystal structure: contains datablock(s) I. DOI: 10.1107/S2056989019011411/nr2075sup1.cif

Structure factors: contains datablock(s) I. DOI: 10.1107/S2056989019011411/nr2075Isup2.hkl

Supporting information file. DOI: 10.1107/S2056989019011411/nr2075Isup3.cml

CCDC reference: 1947007

Additional supporting information:  crystallographic information; 3D view; checkCIF report

## Figures and Tables

**Table 1:** Hydrogen-bond geometry (Å, °)

*D*—H⋯*A*	*D*—H	H⋯*A*	*D*⋯*A*	*D*—H⋯*A*
C4—H4⋯N3^i^	0.95	2.58	3.4819 (18)	159
C12—H12*A*⋯N2^ii^	0.98	2.51	3.373 (2)	147

Symmetry codes: (i) 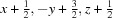; (ii) 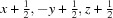.

**Table 2:** Experimental details

Crystal data	
Chemical formula	C_4_H_12_N^+^·C_11_H_5_N_4_^−^
*M*_r_	267.33
Crystal system, space group	Monoclinic, *P*2_1_/*n*
Temperature (K)	150
*a*, *b*, *c* (Å)	10.6043 (5), 9.4168 (4), 16.4423 (7)
β (°)	107.8856 (17)
*V* (Å^3^)	1562.55 (12)
*Z*	4
Radiation type	Mo *K*α
μ (mm^−1^)	0.07
Crystal size (mm)	0.35 × 0.21 × 0.15

Data collection	
Diffractometer	Bruker APEXII CCD
Absorption correction	Multi-scan (*SADABS*; Bruker, 2016 ▸)
*T*_min_, *T*_max_	0.654, 0.747
No. of measured, independent and observed [*I* > 2σ(*I*)] reflections	64536, 6914, 3657
*R*_int_	0.097
(sin θ/λ)_max_ (Å^−1^)	0.810

Refinement	
*R*[*F* ^2^ > 2σ(*F* ^2^)], *wR*(*F* ^2^), *S*	0.067, 0.221, 1.03
No. of reflections	6914
No. of parameters	179
No. of restraints	51
H-atom treatment	H-atom parameters constrained
Δρ_max_, Δρ_min_ (e Å^−3^)	0.50, −0.51

Computer programs: *APEX3* (Bruker, 2018 ▸), *SAINT* (Bruker, 2016 ▸), *SHELXT2017* (Sheldrick, 2015*a* ▸), *SHELXL2018* (Sheldrick, 2015*b* ▸) and *OLEX2* (Dolomanov *et al.*, 2009 ▸).

## supplementary crystallographic information

### Crystal data

**Table:**

C_4_H_12_N^+^·C_11_H_5_N_4_^−^	*F*(000) = 568
*M**_r_* = 267.33	*D*_x_ = 1.136 Mg m^−^^3^
Monoclinic, *P*2_1_/*n*	Mo *K*α radiation, λ = 0.71073 Å
*a* = 10.6043 (5) Å	Cell parameters from 6241 reflections
*b* = 9.4168 (4) Å	θ = 2.6–25.6°
*c* = 16.4423 (7) Å	µ = 0.07 mm^−^^1^
β = 107.8856 (17)°	*T* = 150 K
*V* = 1562.55 (12) Å^3^	Block, light blue
*Z* = 4	0.35 × 0.21 × 0.15 mm

### Data collection

**Table:**

Bruker APEXII CCD diffractometer	3657 reflections with *I* > 2σ(*I*)
Radiation source: sealed X-ray tube	*R*_int_ = 0.097
φ and ω scans	θ_max_ = 35.2°, θ_min_ = 2.5°
Absorption correction: multi-scan (SADABS; Bruker, 2016)	*h* = −17→17
*T*_min_ = 0.654, *T*_max_ = 0.747	*k* = −15→15
64536 measured reflections	*l* = −26→26
6914 independent reflections	

### Refinement

**Table:**

Refinement on *F*^2^	Primary atom site location: structure-invariant direct methods
Least-squares matrix: full	Secondary atom site location: difference Fourier map
*R*[*F*^2^ > 2σ(*F*^2^)] = 0.067	Hydrogen site location: inferred from neighbouring sites
*wR*(*F*^2^) = 0.221	H-atom parameters constrained
*S* = 1.02	*w* = 1/[σ^2^(*F*_o_^2^) + (0.0941*P*)^2^ + 0.374*P*] where *P* = (*F*_o_^2^ + 2*F*_c_^2^)/3
6914 reflections	(Δ/σ)_max_ < 0.001
179 parameters	Δρ_max_ = 0.50 e Å^−^^3^
51 restraints	Δρ_min_ = −0.51 e Å^−^^3^

### Special details

**Table:**

Geometry. All esds (except the esd in the dihedral angle between two l.s. planes) are estimated using the full covariance matrix. The cell esds are taken into account individually in the estimation of esds in distances, angles and torsion angles; correlations between esds in cell parameters are only used when they are defined by crystal symmetry. An approximate (isotropic) treatment of cell esds is used for estimating esds involving l.s. planes.

### Fractional atomic coordinates and isotropic or equivalent isotropic displacement parameters (Å^2^)

**Table:**

	*x*	*y*	*z*	*U*_iso_*/*U*_eq_	Occ. (<1)
N1	0.54341 (16)	0.24960 (18)	0.22446 (9)	0.0608 (4)	
N2	0.36705 (18)	0.37537 (19)	−0.04834 (9)	0.0631 (4)	
N3	−0.17214 (16)	0.90748 (17)	−0.16118 (8)	0.0548 (4)	
N4	−0.29656 (17)	1.11815 (18)	0.04221 (10)	0.0617 (4)	
C1	0.46618 (16)	0.31235 (16)	0.17174 (9)	0.0439 (3)	
C2	0.37143 (14)	0.39111 (14)	0.10849 (8)	0.0375 (3)	
C3	0.37173 (16)	0.37974 (16)	0.02231 (9)	0.0433 (3)	
C4	0.28203 (14)	0.48084 (14)	0.12984 (8)	0.0360 (3)	
H4	0.286927	0.486600	0.188411	0.043*	
C5	0.18676 (15)	0.56214 (15)	0.07251 (8)	0.0391 (3)	
H5	0.178726	0.553107	0.013587	0.047*	
C6	0.10204 (14)	0.65596 (15)	0.09478 (8)	0.0374 (3)	
H6	0.107502	0.663852	0.153364	0.045*	
C7	0.00981 (15)	0.73879 (15)	0.03587 (8)	0.0385 (3)	
H7	0.001958	0.726481	−0.022824	0.046*	
C8	−0.07162 (13)	0.83809 (14)	0.05642 (8)	0.0356 (3)	
H8	−0.068286	0.846208	0.114637	0.043*	
C9	−0.15824 (14)	0.92700 (15)	−0.00276 (8)	0.0363 (3)	
C10	−0.16767 (14)	0.91795 (16)	−0.09079 (9)	0.0400 (3)	
C11	−0.23544 (16)	1.03238 (17)	0.02185 (9)	0.0432 (3)	
N5	0.93087 (11)	0.33415 (12)	0.26390 (7)	0.0352 (2)	
C12	0.90376 (19)	0.17881 (17)	0.25738 (11)	0.0514 (4)	
H12A	0.924319	0.138185	0.314851	0.077*	
H12B	0.810076	0.162583	0.226183	0.077*	
H12C	0.959035	0.133384	0.226798	0.077*	
C13	0.8536 (4)	0.4046 (3)	0.18099 (17)	0.0490 (7)*	0.663 (9)
H13A	0.871124	0.507013	0.184931	0.073*	0.663 (9)
H13B	0.881000	0.364754	0.133987	0.073*	0.663 (9)
H13C	0.758711	0.387889	0.170296	0.073*	0.663 (9)
C14	0.8909 (4)	0.4015 (3)	0.3353 (2)	0.0435 (7)*	0.663 (9)
H14A	0.910320	0.503380	0.337393	0.065*	0.663 (9)
H14B	0.795749	0.387336	0.325245	0.065*	0.663 (9)
H14C	0.940554	0.357500	0.389733	0.065*	0.663 (9)
C15	1.0755 (3)	0.3641 (3)	0.2819 (2)	0.0496 (8)*	0.663 (9)
H15A	1.090359	0.466948	0.285821	0.074*	0.663 (9)
H15B	1.125448	0.319406	0.336004	0.074*	0.663 (9)
H15C	1.105413	0.325613	0.235551	0.074*	0.663 (9)
C13A	1.0743 (7)	0.3428 (10)	0.3146 (7)	0.088 (3)*	0.337 (9)
H13D	1.088217	0.297754	0.370414	0.132*	0.337 (9)
H13E	1.127343	0.293692	0.283871	0.132*	0.337 (9)
H13F	1.101335	0.442639	0.322782	0.132*	0.337 (9)
C14A	0.9016 (9)	0.3989 (6)	0.1798 (3)	0.0563 (16)*	0.337 (9)
H14D	0.920339	0.500938	0.186074	0.084*	0.337 (9)
H14E	0.956852	0.355127	0.148616	0.084*	0.337 (9)
H14F	0.807893	0.384326	0.148001	0.084*	0.337 (9)
C15A	0.8467 (7)	0.3965 (5)	0.3131 (4)	0.0457 (14)*	0.337 (9)
H15D	0.868380	0.351102	0.369345	0.069*	0.337 (9)
H15E	0.863637	0.498739	0.320288	0.069*	0.337 (9)
H15F	0.753021	0.380433	0.281860	0.069*	0.337 (9)

### Atomic displacement parameters (Å^2^)

**Table:**

	*U*^11^	*U*^22^	*U*^33^	*U*^12^	*U*^13^	*U*^23^
N1	0.0710 (10)	0.0648 (10)	0.0455 (8)	0.0213 (8)	0.0162 (7)	0.0058 (7)
N2	0.0830 (11)	0.0716 (10)	0.0389 (7)	0.0087 (9)	0.0247 (7)	−0.0069 (7)
N3	0.0652 (9)	0.0656 (9)	0.0338 (6)	0.0110 (7)	0.0155 (6)	0.0044 (6)
N4	0.0747 (10)	0.0625 (10)	0.0571 (9)	0.0175 (8)	0.0339 (8)	0.0059 (7)
C1	0.0545 (9)	0.0407 (8)	0.0379 (7)	0.0050 (6)	0.0166 (6)	−0.0010 (6)
C2	0.0480 (7)	0.0346 (6)	0.0301 (6)	−0.0007 (5)	0.0121 (5)	−0.0019 (5)
C3	0.0543 (8)	0.0401 (7)	0.0368 (7)	0.0020 (6)	0.0160 (6)	−0.0050 (6)
C4	0.0469 (7)	0.0354 (6)	0.0264 (5)	−0.0043 (5)	0.0123 (5)	−0.0018 (5)
C5	0.0501 (8)	0.0398 (7)	0.0273 (6)	0.0012 (6)	0.0120 (5)	−0.0001 (5)
C6	0.0453 (7)	0.0372 (7)	0.0303 (6)	−0.0028 (5)	0.0125 (5)	0.0000 (5)
C7	0.0468 (7)	0.0407 (7)	0.0286 (6)	0.0004 (6)	0.0128 (5)	−0.0003 (5)
C8	0.0412 (7)	0.0390 (7)	0.0278 (6)	−0.0043 (5)	0.0125 (5)	0.0010 (5)
C9	0.0402 (7)	0.0416 (7)	0.0292 (6)	−0.0010 (5)	0.0138 (5)	0.0011 (5)
C10	0.0414 (7)	0.0454 (8)	0.0335 (6)	0.0037 (6)	0.0118 (5)	0.0038 (5)
C11	0.0491 (8)	0.0486 (8)	0.0353 (7)	0.0024 (6)	0.0179 (6)	0.0043 (6)
N5	0.0400 (6)	0.0364 (6)	0.0307 (5)	−0.0003 (4)	0.0133 (4)	0.0001 (4)
C12	0.0688 (11)	0.0371 (8)	0.0521 (9)	−0.0016 (7)	0.0243 (8)	0.0000 (6)

### Geometric parameters (Å, º)

**Table:**

N1—C1	1.156 (2)	N5—C14A	1.455 (5)
N2—C3	1.1481 (19)	N5—C15A	1.496 (5)
N3—C10	1.1480 (18)	C12—H12A	0.9800
N4—C11	1.148 (2)	C12—H12B	0.9800
C1—C2	1.414 (2)	C12—H12C	0.9800
C2—C3	1.4220 (19)	C13—H13A	0.9800
C2—C4	1.3929 (19)	C13—H13B	0.9800
C4—H4	0.9500	C13—H13C	0.9800
C4—C5	1.382 (2)	C14—H14A	0.9800
C5—H5	0.9500	C14—H14B	0.9800
C5—C6	1.387 (2)	C14—H14C	0.9800
C6—H6	0.9500	C15—H15A	0.9800
C6—C7	1.386 (2)	C15—H15B	0.9800
C7—H7	0.9500	C15—H15C	0.9800
C7—C8	1.3832 (19)	C13A—H13D	0.9800
C8—H8	0.9500	C13A—H13E	0.9800
C8—C9	1.3938 (19)	C13A—H13F	0.9800
C9—C10	1.4225 (18)	C14A—H14D	0.9800
C9—C11	1.422 (2)	C14A—H14E	0.9800
N5—C12	1.4883 (19)	C14A—H14F	0.9800
N5—C13	1.511 (3)	C15A—H15D	0.9800
N5—C14	1.505 (3)	C15A—H15E	0.9800
N5—C15	1.497 (3)	C15A—H15F	0.9800
N5—C13A	1.495 (7)		
			
N1—C1—C2	178.84 (17)	H12A—C12—H12B	109.5
C1—C2—C3	118.35 (13)	H12A—C12—H12C	109.5
C4—C2—C1	121.18 (12)	H12B—C12—H12C	109.5
C4—C2—C3	120.45 (13)	N5—C13—H13A	109.5
N2—C3—C2	176.64 (18)	N5—C13—H13B	109.5
C2—C4—H4	117.4	N5—C13—H13C	109.5
C5—C4—C2	125.15 (12)	H13A—C13—H13B	109.5
C5—C4—H4	117.4	H13A—C13—H13C	109.5
C4—C5—H5	117.6	H13B—C13—H13C	109.5
C4—C5—C6	124.74 (12)	N5—C14—H14A	109.5
C6—C5—H5	117.6	N5—C14—H14B	109.5
C5—C6—H6	118.3	N5—C14—H14C	109.5
C7—C6—C5	123.32 (12)	H14A—C14—H14B	109.5
C7—C6—H6	118.3	H14A—C14—H14C	109.5
C6—C7—H7	117.7	H14B—C14—H14C	109.5
C8—C7—C6	124.69 (12)	N5—C15—H15A	109.5
C8—C7—H7	117.7	N5—C15—H15B	109.5
C7—C8—H8	117.9	N5—C15—H15C	109.5
C7—C8—C9	124.23 (12)	H15A—C15—H15B	109.5
C9—C8—H8	117.9	H15A—C15—H15C	109.5
C8—C9—C10	119.98 (12)	H15B—C15—H15C	109.5
C8—C9—C11	122.28 (12)	N5—C13A—H13D	109.5
C11—C9—C10	117.69 (13)	N5—C13A—H13E	109.5
N3—C10—C9	177.83 (16)	N5—C13A—H13F	109.5
N4—C11—C9	179.3 (2)	H13D—C13A—H13E	109.5
C12—N5—C13	109.12 (15)	H13D—C13A—H13F	109.5
C12—N5—C14	112.07 (14)	H13E—C13A—H13F	109.5
C12—N5—C15	111.28 (15)	N5—C14A—H14D	109.5
C12—N5—C13A	103.6 (4)	N5—C14A—H14E	109.5
C12—N5—C15A	106.9 (2)	N5—C14A—H14F	109.5
C14—N5—C13	108.31 (17)	H14D—C14A—H14E	109.5
C15—N5—C13	109.50 (18)	H14D—C14A—H14F	109.5
C15—N5—C14	106.48 (17)	H14E—C14A—H14F	109.5
C13A—N5—C15A	110.5 (4)	N5—C15A—H15D	109.5
C14A—N5—C12	111.3 (3)	N5—C15A—H15E	109.5
C14A—N5—C13A	113.0 (4)	N5—C15A—H15F	109.5
C14A—N5—C15A	111.2 (3)	H15D—C15A—H15E	109.5
N5—C12—H12A	109.5	H15D—C15A—H15F	109.5
N5—C12—H12B	109.5	H15E—C15A—H15F	109.5
N5—C12—H12C	109.5		
			
C1—C2—C4—C5	179.67 (14)	C5—C6—C7—C8	−176.78 (14)
C2—C4—C5—C6	−176.99 (14)	C6—C7—C8—C9	175.87 (14)
C3—C2—C4—C5	1.4 (2)	C7—C8—C9—C10	0.6 (2)
C4—C5—C6—C7	178.25 (14)	C7—C8—C9—C11	−176.86 (14)

### Hydrogen-bond geometry (Å, º)

**Table:**

*D*—H···*A*	*D*—H	H···*A*	*D*···*A*	*D*—H···*A*
C4—H4···N3^i^	0.95	2.58	3.4819 (18)	159
C12—H12*A*···N2^ii^	0.98	2.51	3.373 (2)	147

Symmetry codes: (i) *x*+1/2, −*y*+3/2, *z*+1/2; (ii) *x*+1/2, −*y*+1/2, *z*+1/2.
